# Transcriptome Analysis Revealed the Possible Reasons for the Change of Ni Resistance in *Rhus typhina* after Spraying Melatonin

**DOI:** 10.3390/plants13101287

**Published:** 2024-05-07

**Authors:** Tongbao Qu, Yinxi Ma, Minqiang Yun, Chunli Zhao

**Affiliations:** College of Forestry and Grassland, Jilin Agricultural University, Changchun 130118, China; qvtb@jlau.edu.cn (T.Q.); mayinxi2903@163.com (Y.M.); michardyi@jlau.edu.cn (M.Y.)

**Keywords:** melatonin, nickel toxicity, *Rhus typhina*, transcriptome analysis, phytoremediation

## Abstract

Melatonin (MT) plays an important role in alleviating the stress of soil heavy metal pollution on plants. However, its ability to improve the tolerance of *Rhus typhina* to Ni stress and its mechanism of action are still unclear. Therefore, MT (0, 50, 100, and 200 μmol·L^−1^) was sprayed on the leaf surface of *R. typhina* seedlings under Ni (0 and 250 mg·kg^−1^) stress to study the differences in growth, physiology, and gene expression. The results showed that exogenous MT could improve the ability of *R. typhina* to resist Ni stress by inhibiting the degradation of chlorophyll and carotenoid, enhancing photosynthesis, and augmenting the activity of antioxidant enzymes. Moreover, 100 μmol·L^−1^ MT could increase the Ni concentration in *R. typhina* seedlings and reduce the translocation factor. Transcriptome analysis showed that MT mainly regulated the expression of related genes in plant hormone signal transduction, starch and sucrose metabolism, and various amino acid metabolism pathways. This study combined physiological and transcriptomic analysis to reveal the molecular mechanism of MT enhancing Ni resistance in *R. typhina*, and provides a new direction for expanding its application in phytoremediation.

## 1. Introduction

Industrial development in urban areas has led to serious heavy metal soil contamination, among which nickel (Ni) is widely of concern as one of the eight major heavy metal pollutants in China [1,2,3]. Elevated concentrations of Ni can damage plant cells, reduce the photosynthetic rate, interfere with enzyme activities, and inhibit nutrient absorption, resulting in a decline in plant biomass [4,5]. Hence, it is imperative to find strategies that enhance plant resistance against Ni [6,7]. Numerous studies have demonstrated that the addition of exogenous substances such as ABA, GA, SA, and JA can augment plant tolerance to heavy metals [8,9,10,11,12,13].

Melatonin (MT) is recognized as a pivotal phytohormone that functions as a molecular cue, modulating gene expression and protein synthesis to enhance plant resistance against various environmental stimuli [14,15,16,17]. The regulation of diverse physiological processes in plants by MT can enhance their resistance to heavy metal toxicity [18,19,20]. The functions encompass the facilitation of seed germination and root growth, optimization of photosynthetic processes as well as the augmentation of osmoregulation [21,22,23]. Several studies have demonstrated that MT can mitigate plant damage induced by Ni stress through modulation of the antioxidant system and scavenging excessive ROS [24,25]. Meanwhile, MT can increase the accumulation of nutrients and improve the resistance of plants to heavy metal stress by enhancing photosynthesis, regulating the gene expression of plant hormone signal transduction (IAA, ABA, GA, etc.), and the starch and sucrose metabolism pathways [26,27,28,29]. The gene expression of various amino acid metabolic pathways can be upregulated by MT. Amino acids can also form stable compounds with heavy metal ions, increasing the accumulation of heavy metals in plants and reducing their damage [30,31,32]. However, the effects of MT vary under different plant and stress conditions.

*R. typhina*, a deciduous shrub or small tree in the Anacardiaceae, possesses excellent characteristics such as rapid reproduction, broad ecological niche, adaptability, drought resistance, salinity tolerance, and barrenness tolerance [33]. Previous studies conducted by our research team demonstrated the exceptional heavy metal resistance of *R. typhina*, specifically toward Pb, Cd, Cu, and Zn [34,35]. This characteristic renders it a highly suitable candidate for soil remediation in areas contaminated with these pollutants. However, whether MT can improve the tolerance of *R. typhina* to Ni and its molecular mechanism are still unclear. Therefore, based on previous studies, we speculated that exogenous MT can alleviate the toxic effect of heavy metal Ni on *R. typhina*, mainly through the following aspects: (1) Melatonin can regulate antioxidant enzymes and photosynthesis, reducing the Ni accumulation of *R. typhina*; (2) Melatonin can regulate related genes of the plant hormone signal transduction pathway (mainly including IAA, ABA, and GA pathways) in *R. typhina* under Ni stress. The findings of this study elucidate the molecular mechanism by which MT enhances the resistance of *R. typhina* to Ni stress, thereby providing a theoretical foundation for advancing the application of *R. typhina* in the phytoremediation of Ni-contaminated soil.

## 2. Materials and Methods

### 2.1. Plant Materials and Growing Conditions

*R. typhina* seeds were collected from the campus of Jilin Agricultural University, Changchun, China (125.410385° E, 43.810433° N). The experiment was conducted in the artificial climate chamber of Jilin Agricultural University from May to October 2023. A mixed soil matrix (garden soil:river sand:turf in a ratio of 2:2:1) was added with 250 mg·kg^−1^ NiCl_2_·6H_2_O for heavy metal passivation for 2 months. The whole and full seeds were placed in boiling water at 100 °C to break dormancy and then soaked for 24 h after the water temperature cooled naturally. Seeds were sown in plastic pots (diameter: 26 cm and depth: 38 cm) containing 0 and 250 mg·kg^−1^ Ni. The pots were then cultured for 90 days under long day (14 h) and light intensity (3500 lx); each treatment was repeated five times.

Ninety-day-old seedlings of *R. typhina* (5–7 leaf stage) were sprayed with 0, 50, 100, and 200 μmol·L^−1^ MT at 20:00 every day, 50 mL each time, for 7 consecutive days. Morphology and related physiological indices were determined. Some samples were frozen in liquid nitrogen and stored at −80 °C for transcriptome sequencing. The experiment comprised eight treatments: Control (CK), 50 μmol·L^−1^ MT (50 MT), 100 μmol·L^−1^ (100 MT), 200 μmol·L^−1^ MT (200 MT), 250 mg·kg^−1^ Ni (250 Ni), 250 mg·kg^−1^ Ni + 50 μmol·L^−1^ MT (250 Ni + 50 MT), 250 mg·kg^−1^ Ni + 100 μmol·L^−1^ MT (250 Ni + 100 MT), and 250 mg·kg^−1^ Ni + 200 μmol·L^−1^ MT (250 Ni + 200 MT).

### 2.2. Measurement of Growth Traits

Five seedlings of *R. typhina* were randomly selected from each treatment, rinsed with deionized water, and dried. Plant height, root length, crown width, and ground diameter were measured using a vernier caliper (Lichen, Shanghai, China), while the fresh weight was determined using an analytical balance (Lichen, Shanghai, China). The fresh plant samples were subsequently placed in an oven to obtain the dry weight after reaching a constant weight [15].

### 2.3. Determination of Ni Concentration in Different Parts

The concentrations of heavy metals were determined by inductively coupled plasma-mass spectrometry (ICP-MS) and inductively coupled plasma-optical emission spectrometry (ICP-OES) [15]. The translocation factor (TF) expresses the displacement of metals from the roots to shoots by the equation:

TF = C_shoot_/C_root_;

C_shoot_: The concentration of Ni in the aboveground part of the plant;

C_root_: The concentration of Ni in plant roots.

### 2.4. Determination of the Chlorophyll, Carotenoid Content, and Gas Exchange Parameters

We selected the fourth fully expanded leaf from the bottom to the top of the *R. typhina*, wiped it surfaces, removed the veins, and cut it into thin strips. Fresh leaf samples weighing 0.1 g were soaked in 10 mL 80% (*v*/*v*) acetone, then shaded extraction for 48 h until the leaves were completely white. After centrifugation at 4000 rpm·min^−1^ for 10 min, the supernatant was taken and the absorbance at 663 nm, 645 nm, and 445 nm was measured by a spectrophotometer (Shanghai Jingke, Shanghai, China). To determine the photosynthetic index, we employed a portable photosynthetic system (Li-6400; LI-COR, Inc., Lincoln, NE, USA). The buffer bottle was consistent with the atmospheric CO_2_ concentration. The left temperature was 25 °C and the relative humidity was 60–70%. After the instrument was stable, it was measured under natural light. The net photosynthetic rate (Pn), transpiration rate (Tr), intercellular CO_2_ concentration (Ci), and stomatal conductance (Gs) of the *R. typhina* leaves were measured at 9:00–11:00 on sunny days [36].

### 2.5. Measurement of Physiological Indicators

Soluble protein (SP) content was determined by the Coomassie brilliant blue method [37]. For each treatment, 0.2 g of fresh leaf sample (the fourth leaf) was weighed and ground into a homogenate. Then, the homogenate was extracted with 10 mL deionized water and centrifuged at 4000 rpm·min^−1^ for 10 min at 4 °C. Each tube of 0.1 mL supernatant was fully mixed with 5 mL 100 μg·mL^−1^ Coomassie brilliant blue G-250 solution for 2 min. The absorbance of the solution was measured at 595 nm through the spectrophotometer (Shanghai Jingke, Shanghai, China).

Malondialdehyde (MDA) content was determined by the thiobarbituric acid method [23]. A total of 0.5 g of fresh leaf samples was ground on ice with 5 mL of 5% (*w*/*v*) trichloroacetic acid (TCA) solution, centrifuged at 4000 rpm·min^−1^ at 4 °C for 10 min, and then the supernatant was collected. Next, the supernatant was mixed with 2 mL TCA solution containing 0.6% TBA. The mixed solution was boiled in a water bath at 100 °C for 30 min. After boiling, it was immediately placed on ice to stop the reaction and centrifuged at 3000 rpm·min^−1^ for 15 min. The absorbance was recorded at 450 nm, 532 nm, and 600 nm through the spectrophotometer (Shanghai Jingke, Shanghai, China).

Superoxide dismutase (SOD) activity was determined by the nitrogen blue tetrazolium photoreduction method [38]. A total of 2.65 mL of the mixture (mixture of 0.05 mol·L^−1^ pH 7.8 PBS 2 mL, 130 mmol·L^−1^ MET 6 mL, 750 μmol·L^−1^ NBT 6 mL, 100 μmol·L^−1^ EDTA-Na_2_ 6 mL, deionized water 5 mL) was added to 0.05 mL enzyme solution and 20 μmol·L^−1^ riboflavin solution 0.3 mL. The reaction temperature was controlled at 25–30 °C, and the mixture was placed in an environment with a light intensity of 4000 lx for 15 min. The absorbance at 560 nm was measured by a spectrophotometer (Shanghai Jingke, Shanghai, China).

### 2.6. RNA Extraction and Gene Expression Assay through the Quantitative Real-Time PCR Method

After conducting comparative analyses of various growth and physiological indicators, we selected four treatment groups for transcriptome sequencing: Control (CK), 250 mg·kg^−1^ Ni (Ni), 100 μmol·L^−1^ MT (MT), and 250 mg·kg^−1^ Ni + 100 μmol·L^−1^ MT (Ni_MT).

The total RNA was extracted from the frozen leaves of *R. typhina* seedlings using TRIzol (Invitrogen, Waltham, MA, USA). RNA purity and quantification were evaluated by a NanoDrop 2000 spectrophotometer (Thermo Scientific, Waltham, MA, USA), and the RNA integrity was assessed using an Agilent 2100 Bioanalyzer (Agilent Technologies, Santa Clara, CA, USA). Next, the transcriptome library was constructed using the VAHTS Universal V6 RNA-Seq Library Prep Kit (Illumina, San Diego, CA, USA) according to the instructions. After the quality of the library was qualified by the Agilent 2100 biological analyzer (Agilent Technologies, Santa Clara, CA, USA), the Illumina Novaseq 6000 (Illumina, San Diego, CA, USA) sequencing platform was used for sequencing to generate a 150 bp double-ended sequence. Transcriptome sequencing and analysis were performed by Shanghai OE Biotechnology Co., Ltd. (Shanghai, China).

### 2.7. Real-Time Fluorescence Quantitative PCR (RT-qPCR)

To assess the accuracy and reproducibility of the sequencing data, we selected significant DEGs for RT-qPCR validation. The internal reference gene chosen for this assay was GAPDH.

### 2.8. Statistical Analysis

All data were statistically analyzed using the SPSS 26.0 (SPSS Inc., Chicago, IL, USA) software package. One-way analysis of variance (ANOVA) was used, and the LSD method was used to compare the significance between treatments (*p* < 0.05). Transcriptome profiling analysis can be found at https://cloud.oebiotech.com/, accessed on 20 March 2024.

## 3. Result and Discussion

### 3.1. MT Alleviated the Hindering Effect of Ni Stress on the Growth and Development of R. typhina

**Figure 1:**
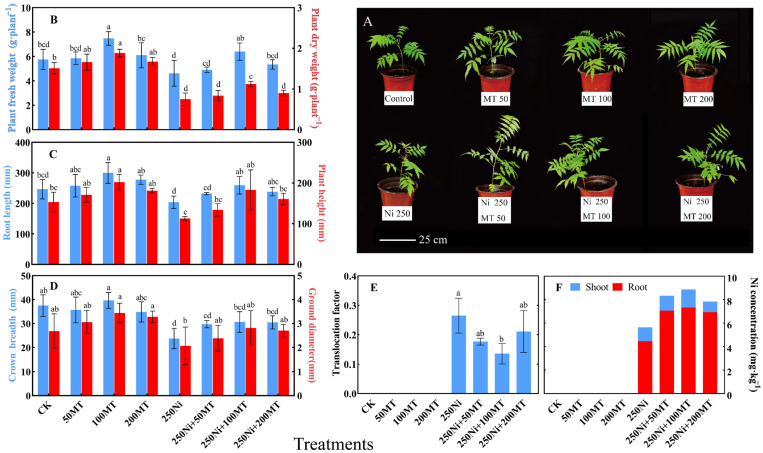
Effects of exogenous MT on the morphological indices and Ni concentration of *R. typhina* seedlings under Ni toxicity. (**A**) Plant growth status; (**B**) Plant fresh weight and dry weight; (**C**) Root length and plant heigh; (**D**) Crown breadth and ground diameter; (**E**) Ni translocation factor; (**F**) Ni concentration in plant. Different letters indicate significant differences between different treatments (*p* < 0.05).

The growth of *R. typhina* seedlings was impeded under Ni stress, while the application of MT enhanced the growth indices of *R. typhina* seedlings under Ni stress (Figure 1). Compared with the CK, the fresh weight, dry weight, plant height, root length, ground diameter, and crown width of the seedlings decreased by 24.44%, 101.33%, 21.12%, 35.81%, 29.80%, and 57.64%, respectively, under Ni stress (*p* < 0.05) (Figure 1B–D). After MT application, the above indices were increased by 38.4%, 49.77%, 27.5%, 62.2%, 36.2%, and 29.0%, respectively (*p* < 0.05), and the effect of 100 μmol·L^−1^ MT treatment was most significant (Figure 1B–D). The findings of this study demonstrated the effective mitigation of Ni stress on plant growth and development by MT; however, it is not a linear relationship where higher concentrations of MT yield more favorable outcomes. Numerous studies have indicated that low concentrations of MT promote plant growth and development, while high concentrations exert negative effects [15,38]. This phenomenon may be attributed to the regulation of plant hormone metabolism and enhanced ethylene synthesis at high MT concentrations, which subsequently inhibits plant growth [39,40,41].

### 3.2. MT Enhanced the Ni Concentration of R. typhina and Reduced the Transport Factor

The concentration of Ni in the roots and shoots of *R. typhina* increased under MT treatment (Figure 1F). Compared to the seedlings without MT treatment, 100 μmol·L^−1^ MT significantly increased the Ni concentration in the root tissue of *R. typhina* by 64.13% (*p* < 0.05). Many studies have shown that applying MT can enhance the synthesis of phytochelatins, increase the concentration of heavy metals in plant roots, and enhance the remediation potential of plants [32,38,42]. Furthermore, MT treatment reduced the translocation factor of Ni in *R. typhina* (Figure 1E). Compared with 250 Ni-treated plants, the translocation factor of 100 μmol·L^−1^ MT decreased by 48.92% (*p* < 0.05). Some studies have shown that MT treatment significantly increased the concentration of heavy metals in the root tissues, while it did not exert a significant impact on the concentration of heavy metals in the aboveground parts of plants [43]. Consequently, this led to a noteworthy reduction in transport factors, which aligns with our experimental findings. The application of MT treatment facilitates the accumulation of Ni in the root tissues and restricts the translocation of Ni from the roots to stems and leaves. This phenomenon can be attributed to the promotion of chelation and compartmentalization processes by MT in the root cells, leading to enhanced fixation and limited transport of heavy metal ions within plants [43,44].

### 3.3. MT Improved Photosynthetic Pigment Content and Gas Exchange Parameters of R. typhina Seedling Leaves under Ni Stress

**Figure 2:**
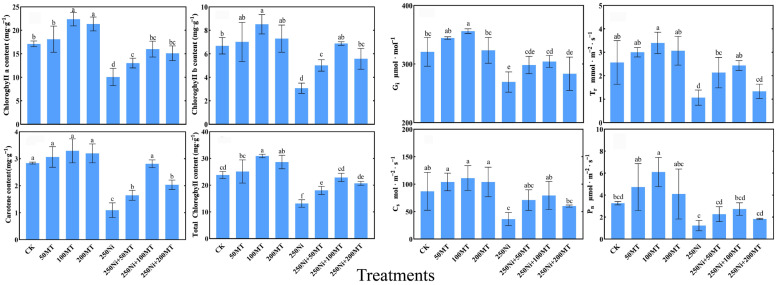
Effects of exogenous MT on photosynthetic indices of *R. typhina* seedlings under Ni toxicity. Different letters indicate significant differences between different treatments (*p* < 0.05).

Our study revealed that exposure to Ni stress significantly reduced the levels of chlorophyll a, chlorophyll b, and carotenoid contents in the *R. typhina* seedling leaves by 41.16%, 54.02%, and 61.50%, respectively, compared to CK (*p* < 0.05) (Figure 2). Notably, the application of exogenous MT effectively mitigated this decline under Ni stress conditions, with the most pronounced effect observed at a concentration of 100 µmol·L^−1^ MT. Specifically, compared to the 250 Ni group, chlorophyll a increased by 58.86%, chlorophyll b increased by 123.65%, and the carotenoids increased by 157.32% (*p* < 0.01). Furthermore, we observed significant reductions in Pn, Tr, Ci, and Gs under Ni treatment; however, supplementation with exogenous MT effectively alleviated these adverse effects on the physiological parameters (Figure 2). In particular, Pn, Tr, Ci, and Gs increased by 121.62%, 128.11%, 12.96%, and 118.35%, respectively, with 100 µmol·L^−1^ MT (*p* < 0.05). Previous studies have consistently reported that Ni stress induces substantial degradation of plant photosynthetic pigments; however, MT has been shown to enhance leaf thickness as well as preserve palisade and sponge tissues along with stomatal size while maintaining intact chloroplast morphology. These protective mechanisms contribute to the preservation of photosynthetic pigments [32,38]. Moreover, the positive impact of MT on photosynthesis may also be attributed to its antioxidant properties [36].

### 3.4. Exogenous MT Alleviated the Physiological Damage of R. typhina under Ni Stress

**Figure 3:**
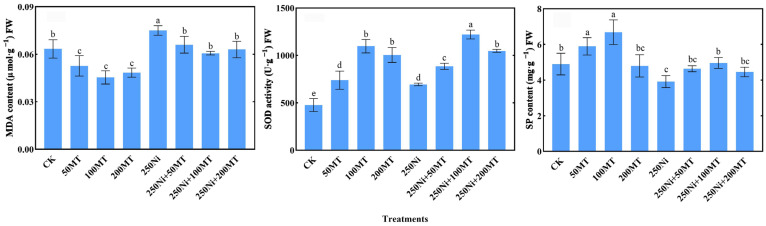
Effects of exogenous MT on physiological indices of *R. typhina* seedlings under Ni toxicity. Different letters indicate significant differences between different treatments (*p* < 0.05).

Exogenous MT reduced oxidative damage by scavenging ROS and enhancing the plant antioxidant defense systems (Figure 3). Compared with the CK, the SP content decreased by 20%, while the SOD activity and the MDA content increased by 45.85% and 18.48%, respectively, in the leaves of *R. typhina* in the 250 Ni group (*p* < 0.05). The excessive accumulation of ROS such as O_2_·^−^ and H_2_O_2_ induced by Ni stress in plants led to elevated MDA levels, indicating that Ni altered the membrane function, resulting in electrolyte leakage, disruption of ion homeostasis, osmotic stress induction, and ultimately, the inhibition of plant growth [21]. After treatment with MT, the activities of antioxidant enzymes in the leaves of *R. typhina* seedlings were significantly enhanced, indicating that MT may effectively reduce the levels of ROS in *R. typhina* by activating reactive oxygen scavenging enzymes and thereby promoting plant growth [15,21,38]. In addition, it suggests that a variety of organic acids and amino acids play important roles in maintaining ROS homeostasis in plants, which requires further investigation [45].

### 3.5. The Potential Molecular Mechanism of MT Regulating the Growth of R. typhina under Ni Stress

#### 3.5.1. Transcriptome Analysis of MT Regulation of Ni Stress

Transcriptome sequencing was performed on 12 leaf samples of *R. typhina* seedlings treated with CK, Ni, MT, and Ni_MT, respectively. A total of 83.75 G clean data were obtained, and the effective data of each sample were above 6.66 G, Q30 > 93.37%, indicating that the sequencing results were reliable and could be used for further analysis. We examined differentially expressed genes (DEGs) between treatments and identified 4089 upregulated and downregulated DEGs in a two-by-two comparison. The number and overlap of DEGs under different treatments were summarized. In the Ni vs. CK comparison, a total of 94 DEGs were identified while in the Ni_MT vs. Ni comparison, there were 1570 DEGs (Figure S1). Notably, the Ni_MT vs. Ni comparison revealed that exogenous MT could regulate a greater number of genes expressed in response to heavy metal stress, as evidenced by 564 upregulated and 1006 downregulated DEGs.

#### 3.5.2. KEGG and GO Enrichment Analyses

**Figure 4:**
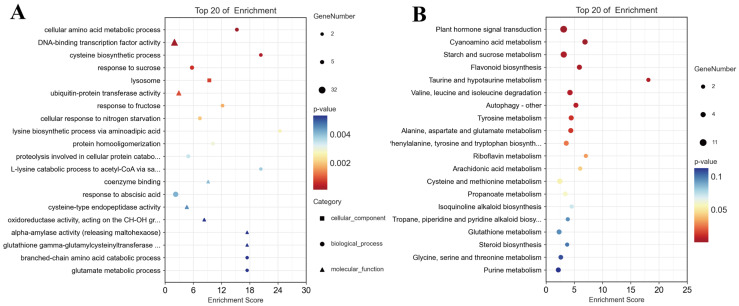
Effects of exogenous MT on differential gene expression of *R. typhina* under Ni toxicity. (**A**) Ni vs. Ni_MT TOP 20 GO enrichment analysis bubble diagram. (**B**) Ni vs. Ni_MT TOP 20 KEGG enrichment analysis bubble diagram. Note: In Figure 4A, “proteolysis involved in cellular protein catabo…” is “proteolysis involved in cellular protein catabolic process”; “L-lysine catabolic process to acetyl-CoA via sa…” is “L-lysine catabolic process to acetyl-CoA via saccharopine”; “oxidoreductase activity, acting on the CH-OH gr…” is “oxidoreductase activity, acting on the CH-OH group of donors, NAD or NADP as acceptor”; “glutathione gamma-glutamyl cysteinyl transferase…” is “glutathione gamma-glutamyl cysteinyl transferase activity”. In Figure 4B, “Phenylalanine, tyrosine and tryptophan biosynt…” is “Phenylalanine, tyrosine and tryptophan biosynthesis”; “Tropane, piperidine and pyridine alkaloid biosy…” is “Tropane, piperidine and pyridine alkaloid biosynthesis”.

To clarify the functional classes of MT-induced DEGs, GO enrichment analyses were performed (Figure 4A). The DEGs in the Ni vs. Ni_MT comparison were assigned to 1204 GO terms, and the identified DEGs were classified into three major GO categories: molecular function, biological process, and cellular component. Among these categories, 58 showed significant enrichment and mainly included the cellular amino acid metabolic process (GO:0006520), DNA-binding transcription factor activity (GO:0003700), cysteine biosynthetic process (GO:0019344), response to sucrose (GO:0009744), lysosome (GO:0005764), ubiquitin-protein transferase activity (GO:0004842), and response to fructose (GO:0009750) (*p* < 0.05).

The functional enrichment pathways were analyzed using the Kyoto Encyclopedia of Genes and Genomes (KEGG) (Figure 4B). The KEGG enrichment analyses revealed that the upregulated genes were predominantly enriched in pivotal pathways including plant hormone signal transduction (ko04075), starch and sucrose metabolism (ko00500), flavonoid biosynthesis (ko00941), alanine, aspartate, and glutamate metabolism (ko00250), and cysteine and methionine metabolism (ko00270), among others (*p* < 0.05). Many studies have shown that exogenous MT can interfere with endogenous plant hormones, upregulate differentially expressed genes in the plant hormone signal transduction pathway, further promote the synthesis of endogenous hormones, and thus enhance the tolerance of plants to heavy metals. Starch and sucrose metabolism play an important role in responding to stress and maintaining plant growth and development [26]. The metabolism of several amino acids such as cysteine is critical in heavy metal chelation [14,29,31,46]. The results of this study also showed that the above pathways were regulated by MT.

#### 3.5.3. MT Regulated a Variety of Amino Acid Metabolic Pathways

**Figure 5:**
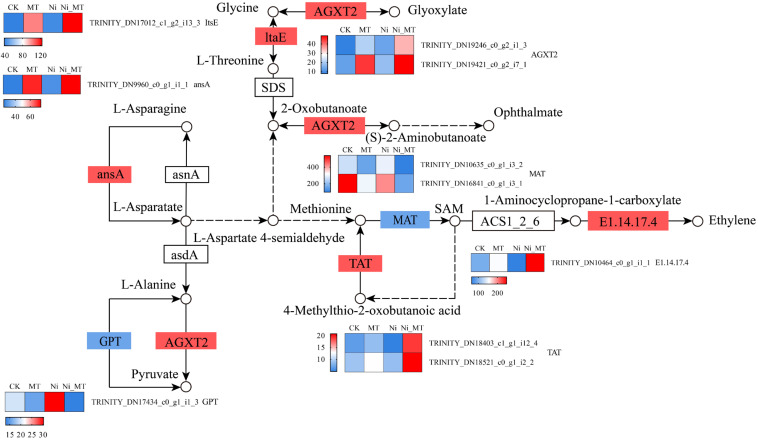
MT regulated the metabolism of many amino acids and gene expression quantity (FPKM) in the Ni vs. Ni_MT comparison. Red represents the upregulation and blue represents the downregulation of this genome.

Amino acids are important for the distribution and transport of heavy metals in plants, and for improving the tolerance of plants to heavy metals [47]. In the Ni_MT vs. Ni comparison, we found gene enrichment related to multiple amino acid metabolic pathways (Figure 5). Three of the main routes included alanine, aspartate, and glutamate metabolism (ko00250), glycine, serine, and threonine metabolism (ko00260), and cysteine and methionine metabolism (ko00270). In the amino acid metabolic pathways, we observed significant regulation of the transcripts of three genes related to alanine, aspartate, and glutamate metabolism (ko00250) treated with exogenous MT. Three genes, which encode L-asparaginase (ASRGL1) and alanine-glyoxylate transaminase (AGXT2), were downregulated under Ni stress. However, these gene expressions were upregulated by MT. The expression of alanine transaminase (GPT) was upregulated under Ni stress, but returned to normal levels after the MT treatment. Exogenous MT upregulated two DEGs, alanine-glyoxylate transaminase (AGXT2) and threonine aldolase (ltaE), in the glycine, serine, and threonine metabolism (ko00260) pathway. In the cysteine and methionine metabolism (ko00270) pathway, two genes encoding S-adenosyl methionine synthetase (metK) and five genes encoding alanine-glyoxylate transaminase (AGXT2), amino cyclopropane carboxylate oxidase (E1.14.17.4), and tyrosine amino transferase (TAT) were upregulated.

Previous studies have demonstrated that MT can induce the upregulation of diverse amino acid metabolic pathways in various species under heavy metal stress, which is consistent with the findings of this investigation [45,47].

#### 3.5.4. MT Regulated Starch and Sucrose Metabolism

**Figure 6:**
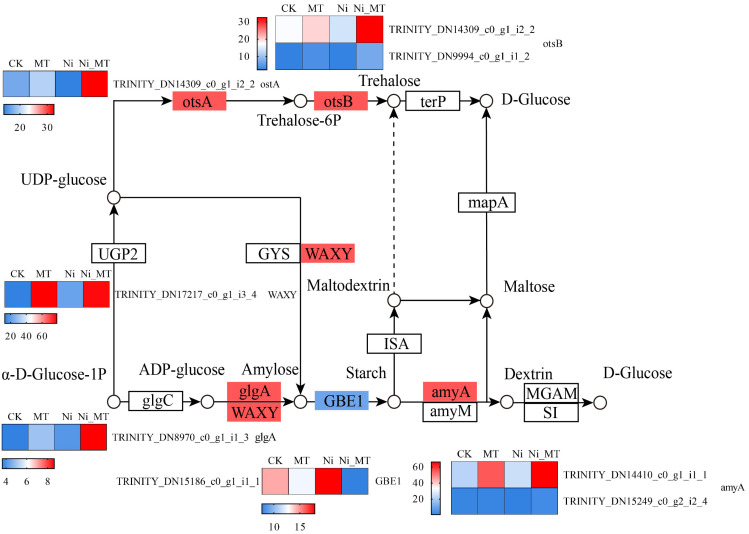
MT regulated starch and sucrose metabolism and gene expression quantity (FPKM) in the Ni vs. Ni_MT comparison. Red represents the upregulation and blue represents the downregulation of this genome.

In Ni vs. Ni_MT comparison, exogenous MT upregulated seven DEGs including one gene encoding trehalose 6-phosphate synthase/phosphatase (TPS), two genes encoding alpha-amylase (AMY), one gene encoding granule-bound starch synthase (WAXY), one gene encoding starch synthase (glgA), and one gene encoding trehalose 6-phosphate phosphatase (otsB). The gene encoding 1,4-alpha-glucan branching enzyme (GBE1) was upregulated under Ni treatment and downregulated under Ni_MT treatment (Figure 6). The findings of several studies have demonstrated that the application of MT leads to an increase in sucrose and starch content, photosynthetic rate, and stress resistance in plants. These results were consistent with our research [29].

#### 3.5.5. MT Regulated Plant Hormone Signal Transduction

**Figure 7:**
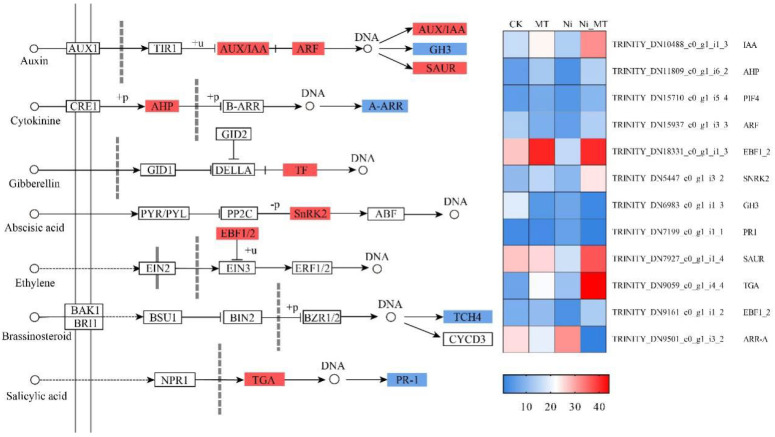
MT regulated plant hormone signal transduction and gene expression quantity (FPKM) in the Ni vs. Ni_MT comparison. Red represents the upregulation and blue represents the downregulation of this genome.

In our study, a total of 18 DEGs were regulated in the plant hormone signal transduction pathway, which were related to seven plant hormone pathways (Figure 7). Auxin is beneficial to the growth and development of plants under heavy metal stress [27]. In addition to the downregulated expression of auxin responsive GH3 gene family (GH3), four DEGs encoding respectively auxin-responsive protein IAA (AUX/IAA), two-component response regulator ARR-A family (ARF), and SAUR family protein (SAUR) were uregulated. Numerous studies have demonstrated the upregulated expression of these genes to impact auxin accumulation, thereby promoting the development of lateral roots and expanding the root surface area for improved nutrient absorption from the soil [10,39]. This also increases the resistance against the toxic effects of heavy metals on plants. Additionally, IAA can stimulate plant root exudation including various metabolites such as phytochelatins (PCs) and organic acids, facilitating enhanced compartmentalization and the chelation of heavy metal ions to improve phytoremediation efficiency in contaminated soils [48]. Cytokinin (CTK) can promote plant growth, physiological activity, and yield in adversity [44]. For the two DEGs in the CTK signaling pathway, histidine-containing phosphor transfer protein (AHP) was upregulated, and the two-component response regulator ARR-A family (A-ARR) was downregulated. This modulation facilitated the transactivation of CTK, promoting cell division and increasing stem thickness. Brassinosteroids (BRs) confer tolerance to various abiotic stresses [49]. In the BR signaling pathway, four genes encoding xyloglucosyl transferase TCH4 (TCH4) were downregulated. The TCH4 transcription factor in this pathway regulated plant height by downregulating its expression, increasing the soluble solids content, and promoting cell elongation [7], which was similar to our findings. MT upregulated the expression of phytochrome-interacting factor 4 (TF) in the GA signaling pathway and two genes encoding EIN3 binding F-box protein (EBF1/2) in the ABA signaling pathway.

Our study revealed that exposure to MT resulted in an upregulation of the ethylene (ETH) signaling pathway and biosynthetic genes, thereby triggering the activation of antioxidant defense mechanisms in plants and subsequently mitigating oxidative stress [46,50,51]. There was a significant upregulation of genes encoding EBF1/2 proteins following MT treatment compared to the Ni stress group. Furthermore, it was discovered that in the cysteine and methionine metabolism (ko00270) pathway, methionine, a precursor of ethylene (ETH) synthesis, was upregulated due to MT-mediated upregulation of amino cyclopropane carboxylate oxidase (E1.14.17.4) expression, resulting in increased ethylene (ETH) biosynthesis to maintain normal plant growth requirements. This confirmed the positive regulatory role of MT in enhancing plant resistance to heavy metals. Salicylic acid (SA) is a phenolic compound that is very important in the plant defense mechanism against environmental stimuli [11,52]. In the SA signaling pathway, the gene encoding transcription factor TGA (TGA) was upregulated. Some studies have shown that MT regulates differentially expressed genes in the SA signaling pathway, which can reduce the damage of stress to plants. This may be because SA can regulate the activity of antioxidant enzymes to re-establish the balance between ROS production and scavenging, reduce the degree of oxidative stress, and enhance the tolerance of plants to heavy metals [53]. Overall, MT can mitigate the harmful effects of Ni on *R. typhina* and improve their resistance to Ni stress by regulating the expression of genes related to the plant hormone signaling pathways.

#### 3.5.6. Validation of DEGs by qRT-PCR

For the accuracy of the experiment, *GAPDH* was selected as the reference gene, and five DEGs, *AGXT2*, *bglB*, *FHY*, *IAA*, and *TGA*, were randomly selected for qRT-PCR. The results showed that the trends of qRT-PCR and RNA-Seq were highly consistent (Figure S2), which confirmed the accuracy and repeatability of the RNA-Seq data in this study.

## 4. Conclusions

**Figure 8:**
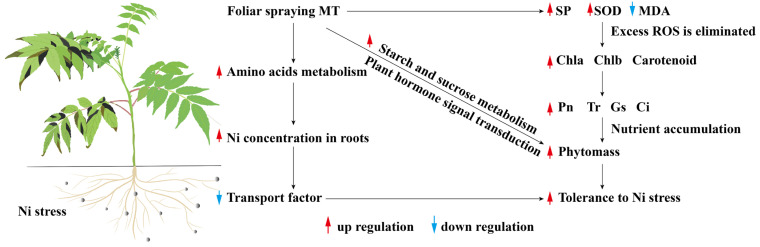
Mechanism hypothesis model of MT improving the tolerance of *R. typhina* seedlings to Ni stress.

An appropriate concentration of exogenous MT can effectively alleviate the significant inhibitory effect of Ni on the dry and fresh weight, plant height, and root length of *R. typhina* seedlings. Through the analysis of its inhibition mechanism, the hypothesis that MT can improve the tolerance of *R. typhina* seedlings to Ni stress was proposed (Figure 8): spraying 100 µmol·L^−1^ MT on the leaves can cause excessive reactive oxygen species in the leaves of *R. typhina* to be removed in time under Ni stress, thereby increasing the content of photosynthetic pigments and enhancing photosynthesis. Moreover, MT promoted *R. typhina* to complex more heavy metal ions by upregulating the gene expression of various amino acid metabolisms, which directly improved the absorption capacity of *R. typhina* to Ni^2+^ in soil, and fixed and separated it in the roots of plants, resulting in the decrease in transport factors and reducing the damage of Ni to *R. typhina* plants. In addition to upregulating IAA, Eth, and CTK, MT also regulates other plant hormone signal transduction pathways such as SA and ABA as well as the gene expression of starch and sucrose metabolism. Through the above methods, MT maintains the phenotypic characteristics of normal plant growth and retains the ornamental value as a garden plant.

## Data Availability

Data are available on request. The data can be made public.

## Supplementary Materials

The following supporting information can be downloaded at: https://www.mdpi.com/article/10.3390/plants13101287/s1. Figure S1: Venn plots of DEGs in the four comparisons. Figure S2: (A) FPKM trends in DEGs analyzed by RNA-Seq. (B) Relative expression trends in DEGs verified by RT-qPCR.
