# Supplementary material for: Transcriptome Analysis Revealed the Possible Reasons for the Change of Ni Resistance in Rhus typhina after Spraying Melatonin
Source: Plants (Basel). 2024 May 7;13(10):1287. doi: 10.3390/plants13101287 (PMC11126081; doi:10.3390/plants13101287)
Supplement: Supplementary file 1 [file plants-13-01287-s001.zip › plants-2975185-supplementary.pdf]

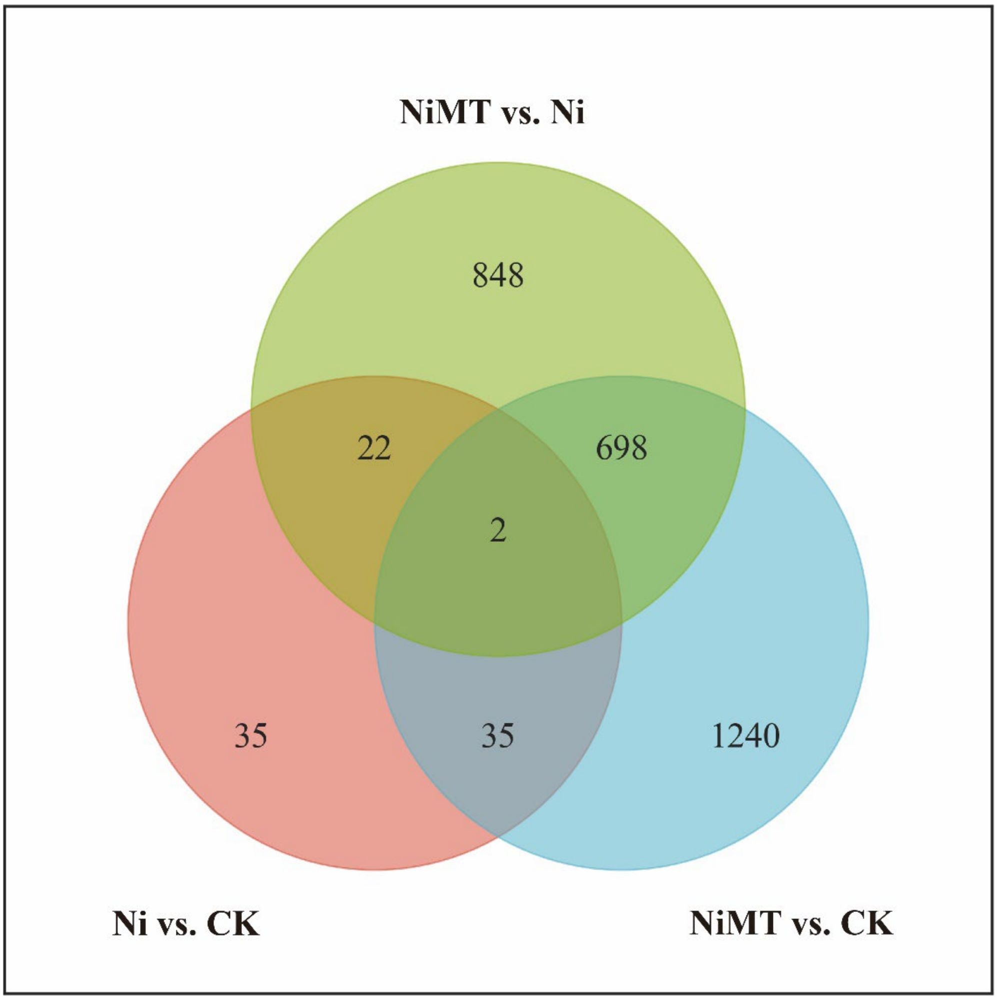

Figure S1. Venn plots of DEGs in the four comparisons.

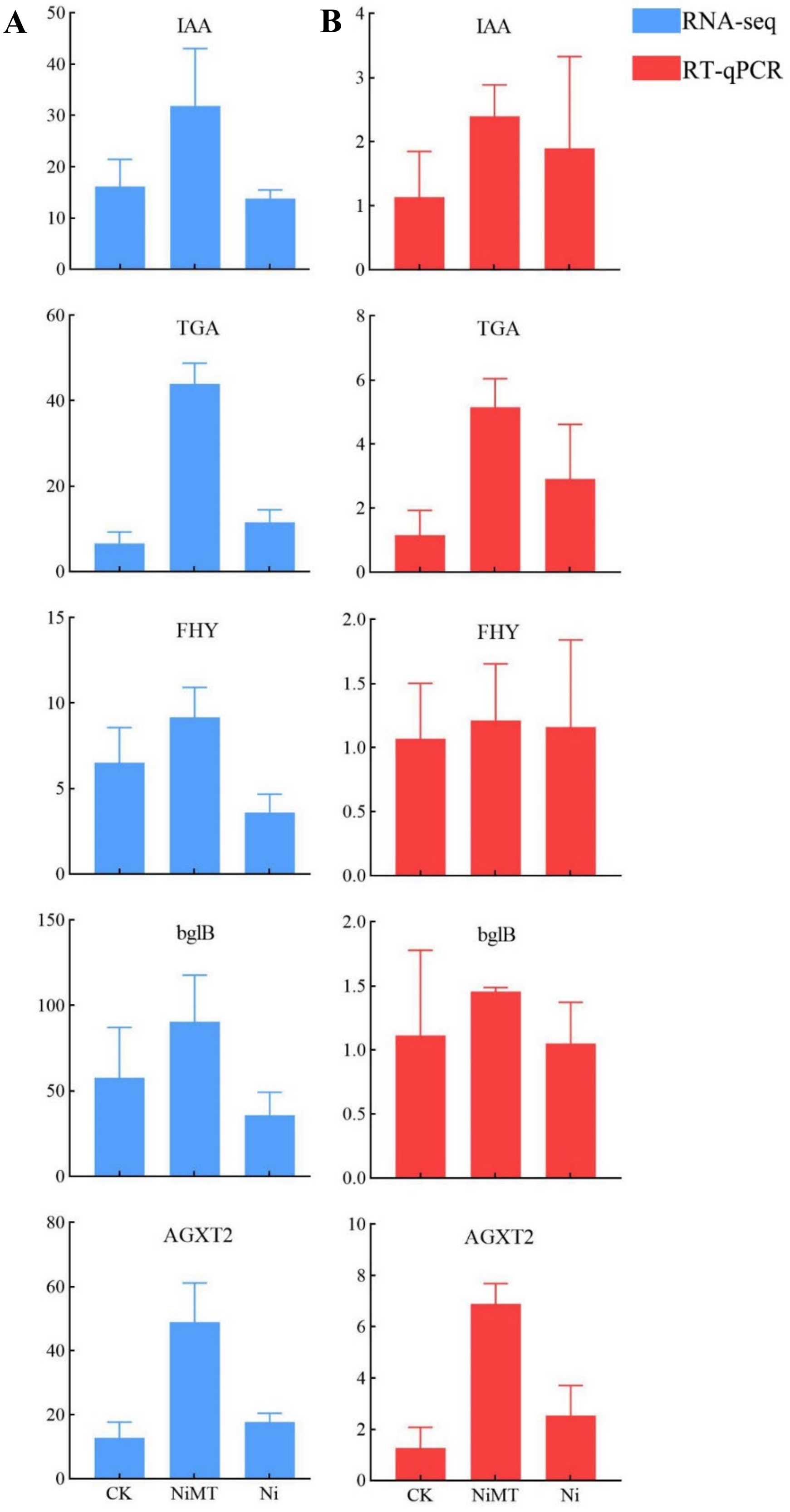

Figure S2. (A) FPKM trends in DEGs analyzed by RNA-seq. (B) Relative expression trends in DEGs verified by RT-qPCR.
